# Beyond the encounter: Predicting multi‐predator risk to elk (*Cervus canadensis*) in summer using predator scats

**DOI:** 10.1002/ece3.8589

**Published:** 2022-02-14

**Authors:** Kara M. MacAulay, Eric G. Spilker, Jodi E. Berg, Mark Hebblewhite, Evelyn H. Merrill

**Affiliations:** ^1^ 3158 Department of Biological Sciences University of Alberta Edmonton Alberta Canada; ^2^ Wildlife Biology Program Department of Ecosystem and Conservation Sciences W. A. Franke College of Forestry and Conservation University of Montana Missoula Montana USA

**Keywords:** *Cervus canadensis*, detection dog, elk, resource selection functions, scat analysis, spatial predation risk

## Abstract

1. There is growing evidence that prey perceive the risk of predation and alter their behavior in response, resulting in changes in spatial distribution and potential fitness consequences. Previous approaches to mapping predation risk across a landscape quantify predator space use to estimate potential predator‐prey encounters, yet this approach does not account for successful predator attack resulting in prey mortality. An exception is a prey kill site that reflects an encounter resulting in mortality, but obtaining information on kill sites is expensive and requires time to accumulate adequate sample sizes.
2. We illustrate an alternative approach using predator scat locations and their contents to quantify spatial predation risk for elk *(Cervus canadensis*) from multiple predators in the Rocky Mountains of Alberta, Canada. We surveyed over 1300 km to detect scats of bears (*Ursus arctos*/*U*. *americanus*), cougars (*Puma concolor*), coyotes (*Canis latrans*), and wolves (*C*. *lupus*). To derive spatial predation risk, we combined predictions of scat‐based resource selection functions (RSFs) weighted by predator abundance with predictions that a predator‐specific scat in a location contained elk. We evaluated the scat‐based predictions of predation risk by correlating them to predictions based on elk kill sites. We also compared scat‐based predation risk on summer ranges of elk following three migratory tactics for consistency with telemetry‐based metrics of predation risk and cause‐specific mortality of elk.
3. We found a strong correlation between the scat‐based approach presented here and predation risk predicted by kill sites and (*r* = .98, *p* < .001). Elk migrating east of the Ya Ha Tinda winter range were exposed to the highest predation risk from cougars, resident elk summering on the Ya Ha Tinda winter range were exposed to the highest predation risk from wolves and coyotes, and elk migrating west to summer in Banff National Park were exposed to highest risk of encountering bears, but it was less likely to find elk in bear scats than in other areas. These patterns were consistent with previous estimates of spatial risk based on telemetry of collared predators and recent cause‐specific mortality patterns in elk.
4. A scat‐based approach can provide a cost‐efficient alternative to kill sites of quantifying broad‐scale, spatial patterns in risk of predation for prey particularly in multiple predator species systems.

## Graphical abstract

This manuscript provides a novel, noninvasive alternative to time/duration‐intensive methods for incorporating a much‐neglected mortality component in quantifying predation risk at the landscape scale. We apply the method to show the implications for understanding the conservation of migratory behavior of a large herbivore in a multi‐predator system.

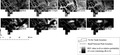

## 1 INTRODUCTION

Large herbivores, like most prey species, make substantial investments in avoiding predation risk (Tolon et al., 2009). Risk of predation influences large herbivore habitat selection, grouping dynamics, and anti‐predator behaviors (Christianson & Creel, 2010; Hebblewhite et al., 2006). As a result, large herbivore prey are often faced with making trade‐offs in pursuing foraging opportunities while avoiding areas of high predation risk (Creel et al., 2005; Hebblewhite & Merrill, 2009). Lima and Dill (1990) established a conceptual model of predation risk by identifying two fundamental components of Holling's (1959) disc equation for the risk of a prey being killed per unit time:

(1) $$P\left(\text{death}\right)=1‐\exp\left(‐\alpha dT\right)$$

where *α* is the probability of encounter and *d* is the probability of death given an encounter during time (T). From the prey's perspective, this approach considers the two main stages of predation and highlights the conditional nature of mortality on attacks. However, it does not explicitly account for how predation risk may vary spatially.

Because spatial data for both predator and prey are increasingly available, predation risk to prey has been related to a predator's abundance, occurrence, and intensity of use or resource selection (Moll et al., 2017; Thaker et al., 2011; Theuerkauf & Rouys, 2008). For example, White et al. (2009) related wolf (*Canis lupus*) density as a metric of predation risk to changes in elk (*Cervus canadensis*) nutrition in Yellowstone National Park during and after wolf recolonization, whereas predation risk from wolves and bears was estimated for a range of prey using RSFs (Gustine et al., 2006). Hebblewhite and Merrill (2007) combined these approaches by weighting RSFs of wolves by their spatial abundance to reflect the importance of the numeric response in predation risk. Although commonly used, these metrics ignore a key component of predation—the probability of death given an encounter (Lima & Dill, 1990). Encounters, however, are extremely difficult to observe directly, (e.g., Eriksen et al., 2009; Whittington et al., 2011). Indirect metrics, like intersections of tracks between predators and prey may adequately measure encounters in space, but not necessarily time (Hebblewhite et al., 2005). As a result, studies have estimated risk of mortality using prey kill sites (Smith et al., 2005). For example, Kauffman et al. (2007) compared wolf kill sites to random locations in Yellowstone National Park to identify landscape features associated with where elk might be killed if visited. Disadvantages in using kill sites is that they often are biased towards large prey that are more readily detected (Bacon et al., 2011; Webb et al., 2008), and in most cases adequate sample sizes take considerable time to accumulate.

An alternative to kill sites is to combine spatial distributions of predators and contents of their scats. Where scat contents reflect primarily predation rather than scavenging events, the location of scat that contains prey reflects the area where a prey encountered a predator and was killed similar to kill sites. In this case, a scat‐based approach to quantifying predation risk may be advantageous over telemetry‐based kill sites because scat surveys are less invasive and may be more cost‐efficient in multi‐predator communities especially when using scat‐detection dogs (Mumma et al., 2017; Wasser et al., 2004). In this paper, we present an approach to quantifying spatial predation risk from bears (*Ursus arctos*/*U*. *americanus*), cougars (*Puma concolor*), coyotes (*C*. *latrans*), and wolves for elk based on predator scats and compare our results to those from kill‐site at point locations and among regions representing different summer ranges of a partially migratory elk herd in the eastern slopes of the Rocky Mountains in Alberta, Canada.

## 2 MATERIALS AND METHODS

### 2.1 Study area

**FIGURE 1:**
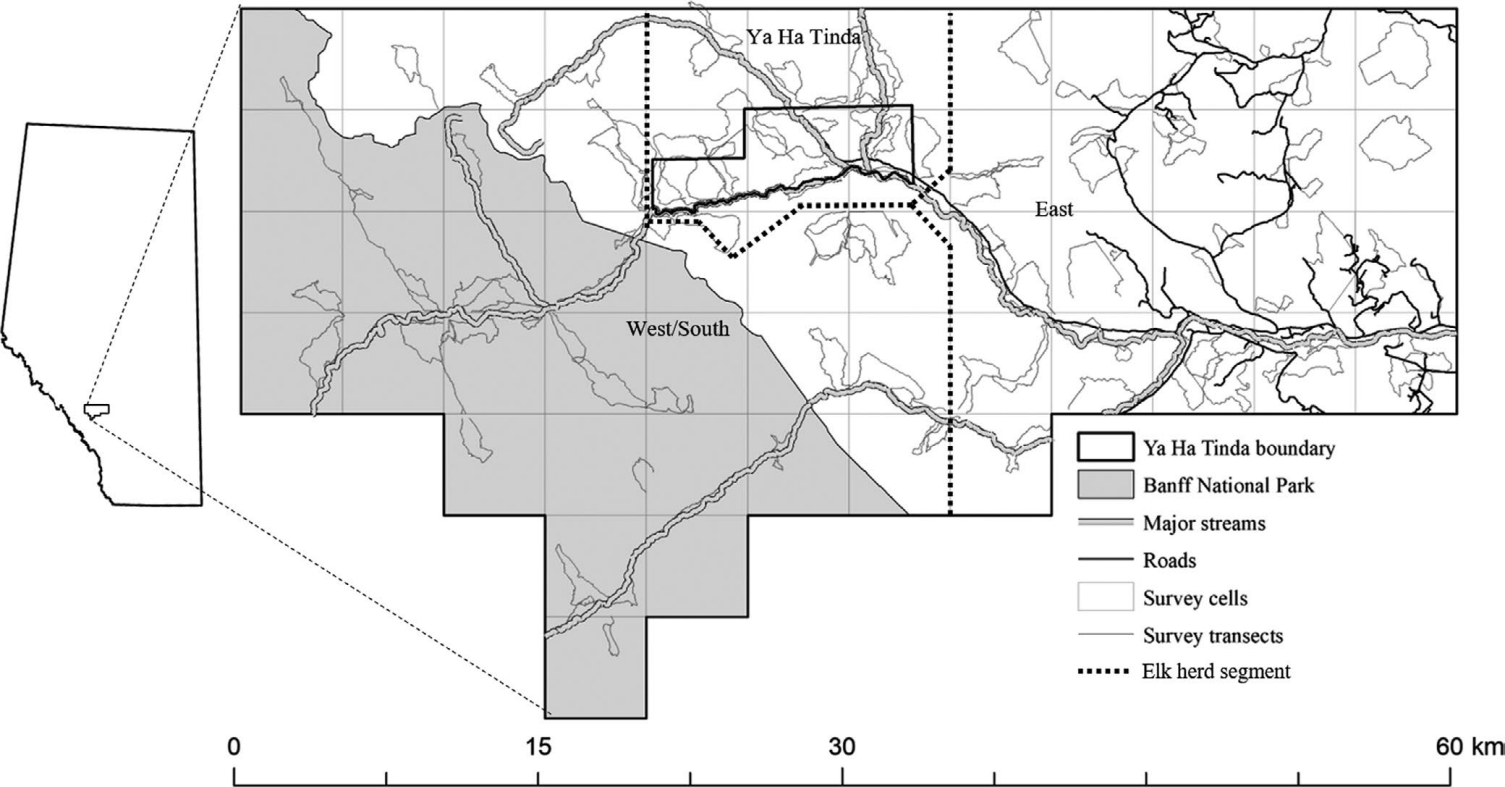
Location of study area along the east slopes of the Rocky Mountain in Alberta, Canada where predator scats were collected along survey transects from 2013 to 2016. Shown are the spatial divisions representing three areas where elk summer including the Ya Ha Tinda ranch and environs (Ya Ha Tinda), the northeastern corner of Banff National Park and environs outside the Park (West/South), and east of Ya Ha Tinda (East)

The study area encompassed the summer range of the partially migratory Ya Ha Tinda elk herd along the eastern slopes of the Rocky Mountains in and adjacent to Banff National Park (Figure 1). The Ya Ha Tinda elk herd has declined by 70% from ~1400 elk counted in 2002 to ~450 elk in 2016 (Berg et al., 2021). Historically, over 90% of the elk population migrated ~50 km west from the Ya Ha Tinda winter range into Banff National Park. More recently, the proportion of resident elk (i.e., elk that remain on the winter range all year round) has increased and more elk are now migrating eastward into areas impacted by forestry (Berg et al., 2021; Hebblewhite et al., 2018). As a result, the migrant to resident ratio has decreased in the last two decades from 3:1 to 1:1 (Berg et al., 2021; Hebblewhite et al., 2006).

High‐elevation bare rock and mixed shrub and herbaceous alpine communities dominated areas >2100 m in the west. Engelmann spruce (*Picea engelmannii*) and subalpine fir (*Abies lasiocarpa*) were the primary high‐elevation conifer landcover, with low‐elevation forests consisting of lodgepole pine (*Pinus contorta*) and white spruce (*P*. *glauca*). Early seral stands (<20‐year stand age) consisted of logged areas (hereafter, “cutblocks”) and post‐fire forest regeneration.

Other ungulates in the area include white‐tailed deer (*Odocoileus virginianus*), mule deer (*O*. *hemionus*), moose (*Alces alces)*, bighorn sheep (*Ovis canadensis*), mountain goats (*Oreamnos americanus*), and feral horses (*Equus caballus*). Wolves naturally recolonized the study area in the mid‐1980s and continue to be relatively stable at least into the early 2000s (Hebblewhite, 2006). Grizzly bears have increased in Alberta (Morehouse & Boyce, 2016), and densities on protected federal land were 2.4 times higher than on provincial lands (Boulanger et al., 2005; Whittington & Sawaya, 2015). Black bear densities were last estimated across Alberta in 1993, and reported as an average of 49 bears per 1000 km^2^ across the five wildlife management units that encompass the study area (Government of Alberta, 2016b). Cougars expanded their range in northern and eastern Alberta since the 1990s (Knopff et al., 2014b). Nothing is known about coyote abundance, although occupancy modeling shows they are ubiquitous across the study area (Steenweg, 2016).

### 2.2 Scat surveys, collection, and analysis

We collected scats using scat‐detection dogs along transects randomly located within a systematic grid of 57 5 × 5‐km cells during 1 July–30 September, 2013–2016. We surveyed 183 km in 2013 as a pilot study, 652 km in 2014 and 405 km in 2015 for scats of all predators, and an additional 82 km in 2016 for cougar scats only, for a total of 1322 km surveyed. Cells in 2014–2015 were resampled but always along a different transect. Due to the difficult topography, survey routes differed from the mapped, random survey routes. A post‐hoc analysis indicated transects transversed elevation classes and landcover types roughly in proportion to that in the study area (i.e., differences <6% for all categories) with the exception of rock/bare ground at high elevation, which differed by 18% (Spilker, 2019). Because radio‐collared elk were rarely found in these areas (<0.01% of 634,004 locations of over 300 collared elk, summers 2002–2016, E. H. Merrill and M. Hebblewhite, unpublished data), we excluded these areas from the resource selection analysis (see below). We sampled transects from July to September because it allowed us to sample scats deposited in the elk calving season (May–June) and before snow that hindered scat detection accumulated at high elevations. Upon scat detection, we recorded age of scat, scat diameter, and physical description to identify scats to species (Elbroch, 2003; Rezendes, 1992; Weaver & Fritts, 1979), and collected DNA on a subsample of scats to assess our species identification accuracy. Age of scats was adapted from Wasser et al. (2004) and included fresh to very old (Spilker, 2019). We omitted old scats judged to be deposited prior to 1 May from all analyses.

Dog‐handler teams (*n* = 4) were trained at Conservation Canines at the University of Washington. We assessed detection of scats by two of the dog‐handler teams who surveyed over 70% of the transects in blind trials where the locations of scats placed in the field were not known to the dog‐handler team. Trials were conducted in conditions and vegetation types similar to those surveyed and dogs were exercised prior to the trials to replicate their level of activity during surveys. Dogs were allowed to search to an approximate effective distance of ~50 m either side of the transect (Long et al., 2007; Reed et al., 2011), and sample scats were recorded as detected or not detected. Each dog handler‐team detected >90% of scats (see Spilker, 2019 for details). We combined grizzly and black bears into one ursid category because we found low accuracy in our ability to discriminate the two based on DNA (<65% correctly classified, *n* = 24; Spilker, 2019).

We analyzed the contents of a subset of scats detected (*n* = 476 of 1118) randomly selected from those detected in 2013–2016 for the presence of elk hair using either macroscopic analysis (*n* = 226) or DNA analysis (*n* = 250). For macroscopic analysis, we randomly selected 20 hairs from each scat, prepared hairs using standard methods (Ciucci et al., 1996), and identified the species based on characteristics of the hairs’ medulla, cuticle scale patterns, and scale margin distance using dichotomous keys (Kennedy & Carbyn, 1981; Moore et al., 1974). Three trained observers who analyzed the scats were subject to blind trials on known hairs, obtaining a minimum of 80% correct classification rate prior to analysis.

DNA was extracted from hair shafts using QIAGEN’s DNeasy Tissue kits (QIAGEN Inc., Valencia, USA). Polymerase chain reaction (PCR) was used to amplify DNA and prey species identification was confirmed via a partial sequence analysis of a hypervariable region of the mitochondrial 16S rRNA gene. This approach identified the most dominant prey species in the scat (i.e., based on the proportion of DNA); mixed samples where there was no dominant species (or equal amounts of DNA from each species) were re‐run with ungulate‐specific primers to determine if elk DNA was present. We compared the presence of elk from the DNA analysis to the macroscopic analysis on the same scats (*n* = 65) based on Area Under the Curve from a Receiver Operating Characteristic curve. We found DNA analysis detected elk present in 88% of the scats where we detected elk macroscopically. It is likely that the 12% of hairs where elk was detected macroscopically but not through DNA analysis is a result of false negatives because of PCR inconsistency (see Mumma et al., 2017). Wildlife Genetics International (Nelson, Canada) performed DNA analyses.

### 2.3 Spatial predation risk

Spatial predation risk (PR_scat_ *_ij_*) reflected the relative risk of an elk dying from a specific predator, *i*, at a location *j*, and was derived as:

(2) $${\text{PR}}_{\text{scat}ij}=P_{\text{pred}ij}*P_{\text{elk}ij}$$

where *P*_pred_ *_ij_* is the scat‐based, resource selection function weighted by predator abundance (see below), and *P*_elk_ *_ij_* is the relative probability of elk being in the predator scat at a location, with *P*_pred_ and *P*_elk_ both being scaled between 0 and 1 by subtracting the minimum value and dividing by the difference between the maximum and minimum value. To estimate total risk (PR_total_) from all predators (bear, cougar, coyote, wolf), we summed the scaled, species‐specific *P*_pred_ *_ij_* values, and similarly scaled the value between 0 and 1.

#### 2.3.1 Relative probability of predator‐scat occurrence

We developed RSFs for predators (Manly et al., 2002), where “used” samples were the locations of predator‐specific scats along transect lines and “available” samples were random locations within a 50‐m × 1.3‐km linear distance centered on the scat. We used this linear distance because it was the average distance moved by black bears in a 24‐h period, which was the shortest 24‐h movement distance among the carnivore species being analyzed, and it standardized availability among predators (Spilker, 2019, Appendix S1). The 50‐m width reflected the estimated distance of scat detection by dogs. We sampled 10 random (available) points within the buffer around each scat location for a density of ~0.8 random points per km^2^, which is just under the 1 random point/km not uncommonly used in telemetry‐based selection studies at the home‐range scale (Hebblewhite and Merrill, 2008; Mumma et al., 2017). We used an exponential RSF model deriving parameters using logistic regression (Johnson et al., 2006). We evaluated subsets of the full set of candidate environment variables and interactions hypothesized for each species using a model selection framework based on AICc to arrive at the best supported RSFs (Burnham & Anderson, 1998). We used a conservative criterion of 4 ΔAICc points in distinguishing competing models to increase confidence that potential explanatory variables would not be excluded during model selection (Burnham & Anderson, 1998). However, in the case of competing models, we followed the principle of parsimony and removed variables where confidence intervals of the parameter overlapped zero.

We selected vegetation, topographic, hydrologic, and anthropogenic features as model inputs (Table 1) because they have been associated with predator occurrence based on previous studies telemetry studies (Knopff et al., 2014a; Nielsen et al., 2002; Whittington et al., 2005). We measured vegetation based on landcover as derived from TM Landsat Imagery where burned areas were ≤14 years old since burning (Hebblewhite, 2006), and cutblocks ≤20 years since harvest (Visscher & Merrill, 2009), vegetation “greenness” from the Normalized Difference Vegetation Index (NDVI). These variables were quantified as the mean or proportion of 30 × 30‐m pixels within a 1.3‐km radius (5.3‐km^2^) buffer around a scat or random location (Table 1). This buffer size reflected the average daily movement of black bears, which was the shortest distance of all the predator species (see Appendix S1). Forest edge was based on a 30‐m buffer of conifer or mixed‐deciduous forest with any other landcover type. Topographic features included slope, elevation, and terrain ruggedness and values for a 30x30‐m cell were derived from a Digital Elevation Model (Table 1). Waterways were measured as the shortest distance (km) to the nearest stream, river or lake feature. Anthropogenic features included distance to motorized roads/trails and nonmotorized trails, as well as motorized and nonmotorized road density (km/km^2^). Use of nonmotorized trails by a predator was input as a categorical variable where scats or random points within 30 m of a trail were considered on‐trail and those further than 30 m were off‐trail.

**TABLE 1:** List of covariates used in scat‐based resource selection functions of predators (*P*_pred_), models predicting elk presence in scats (*P*_elk_), and models predicting kill‐site‐based predation (PR_kill_)

Variable	Code	Units	Analysis unit size	Source of Data	Year of data	Model
Distance to forest edge	distedge	km	–	Derived from TM Landsat imagery from ABMI^a^	2014	*P*_pred_, *P*_elk_, PR_kill_
Forest edge density	edgedens	%	*P*_pred_ – 5.3 km^2^ *P*_elk_ – Appendix S1	Derived from TM Landsat imagery from ABMI^a^	2014	*P*_pred_, *P*_elk_
Distance to stream	distwater	km	–	AltaLIS^b^	1996	*P*_pred_, PR_kill_
Stream density	waterdens	km/km^2^	Appendix S1	AltaLIS^b^	1996	*P*_elk_
Vegetation greenness (NDVI)^c^	ndvi	−1 to 1	5.3 km^2^	MODIS	2013–2016	*P*_pred_
Herbaceous forage biomass	herbfg	g/m^2^	*P*_elk_ – Appendix S1 PR_kill_ – 250m^2^	Berg et al. (2021)	2013–2016	*P*_elk_, PR_kill_
Conifer forest cover	conifer	%	*P*_pred_ – 5.3 km^2^ *P*_elk_ – Appendix S1	Derived from TM Landsat imagery	2016	*P*_pred_, *P*_elk_
Deciduous‐mixed forest cover	decid	%	*P*_pred_ – 5.3 km^2^ *P*_elk_ – Appendix S1	Derived from TM Landsat imagery	2016	*P*_pred_, *P*_elk_, PR_kill_
Herbaceous cover	herb	%	*P*_pred_ – 5.3 km^2^ *P*_elk_ – Appendix S1	Derived from TM Landsat imagery	2016	*P*_pred_, *P*_elk_
Shrub cover	shrub	%	*P*_pred_ – 5.3 km^2^ *P*_elk_ – Appendix S1	Derived from TM Landsat imagery	2016	*P*_pred_, *P*_elk_
Burn	burn	%	*P*_pred_ – 5.3 km^2^ *P*_elk_ – Appendix S1	Derived from TM Landsat imagery	2016	*P*_pred_, *P*_elk_
Cutblocks	cutblk	%	*P*_pred_ – 5.3 km^2^ *P*_elk_ – see Appendix S1	ABMI^a^ ‐ Human Footprint Inventory	2014	*P*_pred_, *P*_elk_
Elevation	elev	m	*P*_pred_ – 5.3 km^2^ *P*_elk_ – Appendix S1	Derived from 20K Digital Elevation Model	2009	*P*_pred_, *P*_elk_
Slope	slope	0–90˚	*P*_pred_ – 5.3 km^2^ *P*_elk_ – Appendix S1	Derived from 20K Digital Elevation Model	2009	*P*_pred_, *P*_elk_
Terrain ruggedness	rugg	0–1	*P*_pred_ – 5.3 km^2^ *P*_elk_ – Appendix S1	Derived from 20K Digital Elevation Model	2009	*P*_pred_, *P*_elk_, PR_kill_
Non‐motorized trail use	trailuse	0/1	30m^2^	AltaLIS^b^	2014	*P*_pred_
Distance to motorized trail/road	distroad	km	–	AltaLIS^b^	2014	*P*_pred_, *P*_elk_
Distance to nonmotorized trail	disttrail	km	–	AltaLIS^b^	2014	*P*_pred_, *P*_elk_
Motorized road density	roaddens	km/km^2^	*P*_pred_ – 5.3 km^2^ *P*_elk_ – Appendix S1	AltaLIS^b^	2014	*P*_pred_, *P*_elk_
Nonmotorized trail density	traildens	km/km^2^	*P*_pred_ – 5.3 km^2^ *P*_elk_ – Appendix S1	AltaLIS^b^	2014	*P*_pred_, *P*_elk_
Elk resource use	RUF	0–1	See Appendix S1	MacAulay (2019)	2013–2016	*P*_elk_
Open canopy	open	%	*P*_elk_ – see Appendix S1 PR_kill_ – 250 m^2^	Derived from TM Landsat imagery from ABMI^a^	2014	*P*_pred_, PR_kill_

Note

The resolution of all variables unless otherwise stated is 30 × 30‐m (900‐m^2^). When analysis units differ by species, source Appendix in Supplemental Information is referenced.

^a^ Alberta Biodiversity Monitoring Institute (www.abmi.ca).

^b^ AltaLIS Alberta Open Data (www.altalis.com).

^c^ Resolution size is 250 × 250‐m.

We evaluated the spatial prediction of scat‐based wolf and bear RSFs by comparing the predicted scat‐based RSF values to RSF values derived from locations of GPS‐collared wolves (Hebblewhite & Merrill, 2007) and grizzly bears (Nielsen et al., 2002). The telemetry‐based wolf RSF was derived from summer locations of 15 GPS‐collared wolves in five packs for summers 2002–2004 (Hebblewhite & Merrill, 2007). The telemetry‐based grizzly bear RSFs were derived from nine bears (6 females, 3 males; Nielsen et al., 2002) for the seasons of hypophagia (15 April–14 June), early hyperphagia (15 June–7 August), and late hyperphagia (8 August to denning) in 2004. We updated both wolf and bear telemetry‐based RSFs for years 2014–2015 to account for landcover changes (such as new timber harvest and fires, see details in Berg et al., 2021). We used Pearson rank correlations at a random set of points (*n* = 1000) distributed across the study area after removing areas of high elevations (>2000 m), bare rock, or ice. Telemetry‐based grizzly bear and wolf RSF values were scaled from 0 to 1, then aggregated into 10 classes based on approximately equal area representation (Nielsen et al., 2002).

Because encounter risk (*P*_pred_) is a function the likelihood of occurrence and the spatial abundance of the wolves, we included an effect for spatial abundance of wolves and bears but not cougars and coyotes because we had data available only for these two species. We weighted the wolf RSF by a spatial probability density function (PDF) based on wolf pack size and annual kill rates (Berg et al., 2021; Hebblewhite & Merrill, 2007). Weighting predation risk by number of bears was not as straight forward because we had abundance estimates only of grizzly bears yet we combined grizzly and black bear scats to derived a single bear RSF. Based on a random sample of bear scats with known identity from DNA, 85% of bear scats found were from grizzly bears (Spilker, 2019). As a result, we weighted the bear RSF only by estimates of grizzly bear densities. Grizzly bear density was 2.4 times higher within Banff National Park compared to outside Banff National Park (Government of Alberta, 2016a; Whittington & Sawaya, 2015). To avoid an abrupt change in density along the border between the Banff National Park and adjacent Provincial public lands, following Berg et al., (2021) who used telemetry‐based RSFs, we smoothed the PDF values along the border using a 12.9‐km moving window, with the size corresponding to the average home range for local grizzly bears (Nielsen et al., 2002).

#### 2.3.2 Relative probability of elk occurrence in predator scats

To predict the relative probability of elk occurrence in a predator‐specific scat at a specific location (*P*_elk_ *_ij_*), we contrasted locations of scats containing elk (*n* = 157: 24 bear, 75 wolf, 42 coyote, and 16 cougar) to locations of a larger set of scats (*n* = 870: 257 bear, 363 wolf, 223 coyote, and 27 cougar) from the same predator species but not analyzed for prey contents, similar to a use/available design (Manly et al., 2002). By using scat locations as our available locations (rather than random locations), we controlled for the influence of landscape features on where predator scats were located per se. We used a model selection approach based on a ΔAIC_c_ > 4 to determine the model with the most support. In the event of competing top models, we followed the principle of parsimony and removed variables where confidence intervals of the parameter overlapped zero. We determined model covariates as the density or distance to linear features (i.e., forest edge, roads, trails, waterways), percent of landcover types, or mean value for continuous variables within a buffer around a scat whose radius was derived from the mean gut passage time and movement per day (i.e., 3‐km for wolf, 1.5‐km for bear, and 2‐km for cougar and coyote, Table 1, Appendix S1). Green herbaceous biomass (g/m^2^) at the peak of the growing season (7 August) was derived from a general linear model based on field sampling of 983 sites across the summer extent of the Ya Ha Tinda elk herd (Hebblewhite et al., 2008), and updated for changes in forage availability caused by climate, timber harvest, and fires (Berg et al., 2021).

Because elk must use the area to be found in the scat, we also quantified relative intensity of elk use using a population‐level resource utilization function (RUF; Marzluff et al., 2004) as a model input. We built a utilization distribution from 6‐hour GPS relocations of 66 adult female elk during 2013–2016 ($\bar{x}$ = 359 relocations per individual from 1 May to 30 September). We used predicted RUF instead of the output of the utilization distribution directly, because GPS‐collared elk represented only ~16% of the population and did not inhabit all the areas surveyed by our scat transects. We opted for an elk RUF rather than a RSF because unlike with the predators where we had a limited number of scats/species we had many summer GPS locations for many elk in an area and decided the relative intensity of use would better reflect elk encounters. Variable inputs in the RUF included herbaceous and total (herbaceous and shrub) forage biomass, herbaceous land cover (Hebblewhite et al., 2008), distance to nearest forest edge, burned vegetation (Hebblewhite, 2006), wolf predation risk (Hebblewhite & Merrill, 2007), and grizzly bear predation risk (Nielsen et al., 2002). We used the *ruf* package in R 2.13 (Marzluff et al., 2004) to obtain resource‐use coefficients. To derive the RUF, we ranked models using AIC calculated from the Matérn maximum log‐likelihood estimate, with a cut‐off of ΔAIC = 4 to determine the model with most support (Burnham & Anderson, 1998). The top model showed elk use of areas with high herbaceous forage biomass, burned areas, areas further from forest edge, and in areas of high wolf and low grizzly bear selection; herbaceous biomass performed better as a metric for forage biomass than total (both shrub and herbaceous) biomass (Appendix S2). The mean RUF value at 1000 telemetry points was significantly higher than the mean RUF value at 1000 random points (*t* = 1.96, *df* = 1,998, *p* < .001), indicating support for the elk resource utilization model (see MacAulay, 2019 for more details).

#### 2.3.3 Scat‐based versus kill‐site predation risk

We compared the predictions of total predation risk from all predators (PR_total_; i.e., sum of PR_scat_ for each predator and scaled from 0 to 1) to predictions of predation risk derived from known kill sites of collared and uncollared elk (*n* = 104) determined to be killed by predators (i.e., not scavenged, see Barber‐Meyer, 2006); between 2002 and 2016 (PR_kill_; 42 bear, 16 cougar, 37 wolf, 9 unknown predator). Following methods established in Kauffman et al. (2007), we derived kill‐site models by comparing features at 104 locations of elk kill sites (1) to 20 random points (0) each using conditional logistic regression. Random points were generated within 13.2 km of each kill site, to account for the largest average movement per day of the predators (i.e., cougar; Dickson et al., 2005; Laundré, 2005). Landscape variables were the percentage or mean value within a 250‐m buffer around the kill sites or random location, to account for variation in where the kill occurred relative to the location of the elk carcass. We used inverse frequency weighting to account for differences in number of kills by each predator species. We evaluated models with AIC_c_ and used the best‐supported model to predict predation risk (PR_kill_) to elk (Appendix S6), assuming that the sample of elk mortalities reflected the relative predator‐specific kill rates. Predictions of kill‐site predation risk were similarly scaled between 0 and 1 as above. We conducted Pearson rank correlations between predictions from scat‐based and kill‐site models at 1000 random points, but graphically presented smoothed graphs based on the mean risk value of 10 equal‐area bins.

## 3 RESULTS

### 3.1 Scat‐Based predator resource selection functions

We used detections of 373 bear, 42 cougar, 223 coyote, and 470 wolf scats to determine RSFs. For bears, there was equal support (ΔAIC < 4) for two models that differed by the inclusion of percent of area in cutblocks. We chose the model including cutblocks as the top model because the confidence limit of the coefficient for cutblocks did not encompass zero (Appendix S3). Bears selected against conifer forests, motorized trails, and roads, and for areas with cutblocks, high NDVI, steep slopes, and nonmotorized trails, particularly when farther from areas with motorized trails (Table 2).

**TABLE 2:** Beta coefficients (*β*) and upper and lower 95% confidence intervals (CI) for the top scat‐based resource selection functions (*P*_pred_) for four predators in the eastern slopes of the Rocky Mountains, Alberta, Canada, 2014–2016

Species	Variable	*β*	95% CI	
			Lower	Upper
Bear	Conifer forest	−0.71	−1.23	−0.19
	Cutblocks	0.84	0.23	1.45
	NDVI	0.0002	0.00007	0.00033
	Slope	0.02	0.019	0.039
	Nonmotorized trail use	0.86	0.41	1.31
	Distance to motorized trail/road	0.00005	0.00003	0.00007
	Nonmotorized trail use*Distance to motorized trail/road	0.00005	0.00001	0.00009
Cougar	Conifer forest	−1.92	−3.38	−0.46
	Forest edge density	8.39	1.12	15.56
Coyote	Shrub	2.63	0.21	5.05
	Slope	−0.05	−0.08	−0.02
	Nonmotorized trail use	1.62	1.27	1.97
	Distance to motorized trail/road	0.00006	0.00004	0.00008
Wolf	Distance to streams	−0.0001	−0.00015	−0.00005
	Cutblocks	−2.47	−4.48	−0.46
	Slope	−0.04	−0.06	−0.02
	Nonmotorized trail use	1.29	0.99	1.59
	Distance to motorized trail/road	0.00005	0.00004	0.00006

There was equal support for four models describing resource selection for wolves, which differed based on the inclusion of either edge, grassland, or both (Appendix S3). We chose the model excluding grassland and edge as the top model because their confidence limits overlapped zero (Table 2). Wolf scats most likely occurred near waterways, on gentler slopes and nonmotorized trails, but farther from motorized trails. Equally supported models for coyotes included areas with gentler slopes and vehicle‐restricted trails, and areas farther from vehicle‐permitted trails (Table 2). We retained proportion of shrub cover because its confidence limits did not overlap zero. Cougar scats were more likely in areas with less conifer forest cover and high edge density because the confidence limits of only these two variables did not overlap zero (Table 2; Appendix S3).

Scat‐based and telemetry‐based RSF values were correlated for wolves (*r*_s_ = .18, *p* < .001, *n* = 1000) and bears (*r*_s_ = .17–0.25, *p* < .001) depending on season (hypophagia, early hyperphagia, and late hyperphagia), but the relationships were not strongly linear (Appendix S4). Nevertheless, scat‐based values increased as telemetry‐based values increased. When aggregated into 10 bins, rank correlations of the mean RSF bin values indicated much higher correspondence (wolf: *r*_s_ = .92, *p* < .0001; grizzly bear: *r*_s_ = .68, *p* < .0001).

**FIGURE 2:**
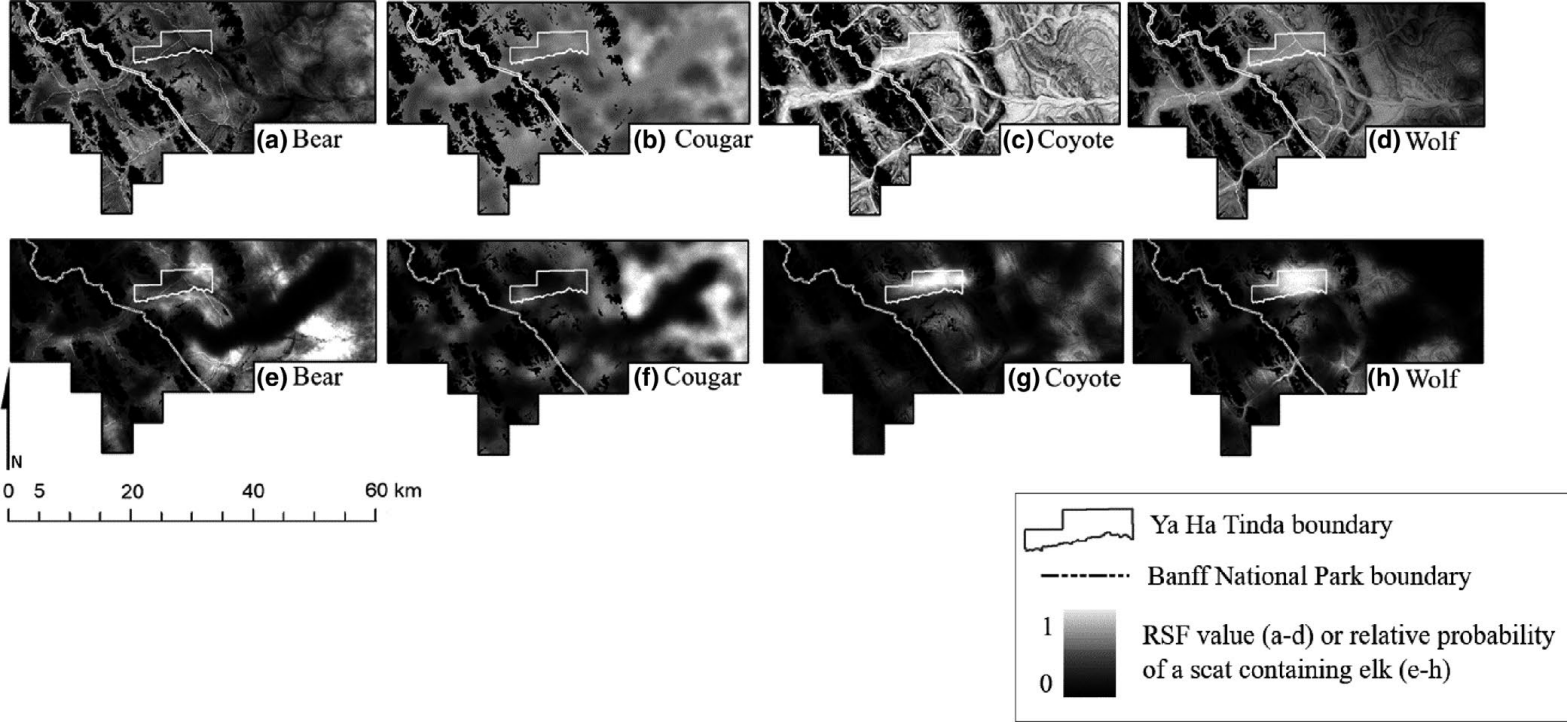
Spatial predictions from scat‐based predator resource selection functions (P_pred_, a–d) and relative probability of elk occurrence in predator scat (P_elk_, e–h) in the study area along the eastern slopes of the Rocky Mountains, Alberta, Canada. Values were scaled between 0 and 1

*P*_pred_ from bears indicated that elk were more likely to be encountered in the western portion of the study area along river drainages than in the eastern portion of the study area (Figure 2a, Table 4). *P*_pred_ was highest for wolves but low for bears (Figure 2a,d, Table 4) on the resident elk home range round the Ya Ha Tinda compared to other summer ranges. Cougar *P*_pred_ increased from the west (low) to east (high), corresponding with more forest edge in the forest‐managed lands (Figure 2, Table 4). Coyote *P*_pred_ was fairly consistent across the study area (Figure 2c, Table 4).

### 3.2 Elk presence in predator scats

We analyzed the contents of 476 scats (130 bear, 33 cougar, 114 coyote, and 199 wolf); 226 were analyzed via macroscopic analysis and the remainder via DNA analysis. Elk was equally found in coyote (36% of scats), wolf (38%), and cougar (46%) scats collected from across the study area (Wilcoxon Rank Sum test; all pairwise *p* ≥ .27). Bear scats contained elk less frequently compared to the three other predators (19%, all pairwise *p* < .001).

We did not use herbaceous forage biomass and elk resource use (RUF) in the same models for predicting elk in a scat, nor did we use slope, elevation, and ruggedness in the same models because they were highly correlated (*r* > .60). The best‐supported, predator‐specific models predicting elk presence in a scat most consistently included the positive effect of herbaceous biomass, except for cougar. Inclusion of other variables depended on the predator species (Appendix S5). For bears, the top model included the positive effect of herbaceous biomass, and negative effect of open cover type (Table 3). Distance to trail, road density, and rugged terrain were also in the top four competing models (Appendix S5), but we used the most parsimonious (Table 3). For wolves, we selected the model including only herbaceous biomass, terrain ruggedness, and percent of area covered by deciduous forest (Table 3) because the confidence limits of the beta coefficient for burns included zero (Appendix S5).

**TABLE 3:** Beta coefficients (β) and lower and upper confidence intervals (CI) for the top predator‐specific models predicting the relative probability of elk occurrence in scat (*P*_elk_) based on Akaike's Information Criterion corrected for small sample sizes (AIC_c_) for four predators along the eastern slopes of the Rocky Mountains, Alberta, Canada, 2013–2016

Species	Variable	*β*	95% CI	
			Lower	Upper
Bear	Herbaceous forage biomass	0.06	0.03	0.10
	Open cover	−4.83	−8.58	−1.63
Cougar	Forest edge density	1.25	0.31	2.49
Coyote	Herbaceous forage biomass	0.050	0.030	0.070
	Distance to streams	0.00032	0.00006	0.00058
	Motorized road/trail density	−0.88	−1.83	−0.13
Wolf	Herbaceous forage biomass	0.21	0.16	0.27
	Terrain ruggedness	0.85	0.53	1.19
	Deciduous‐mixed forest	−36.29	−56.85	−18.25

For elk presence in coyote scat, we selected the model that included the positive effects of herbaceous biomass, distance to water, and the negative effect of road density (Table 3). Highest uncertainty was found in the models of elk presence in cougar scats largely because of low sample size. There were five models with equal support, where single variable models had lower AIC_c_ values compared to models consisting of ≥2 variables (Appendix S5). We selected the model with forest edge density as our top model based on parsimony, as it had a relatively high model weight, and had the same explanatory power (ΔAIC < 2) as including distance to nearest trail.

*P*_elk_ of wolves and coyotes averaged highest on the summer range of residents at the Ya Ha Tinda (Figure 2g,h, Table 4). *P*_elk_ of bears was low in the summer ranges in the western portion of the study, and similar on the resident summer range at the Ya Ha Tinda and in the eastern portion of the study (Figure 2e, Table 4), whereas *P*_elk_ of cougar was highest in eastern part of the study area (Figure 2f, Table 4).

**TABLE 4:** Mean ± standard deviation of predicted (30 m^2^) values across space for scat‐based, weighted resource selection functions (*P*_pred_), relative probability of elk being present in scat (*P*_elk_), and scat‐based predation risk (PR_scat_) based on Equation 2 for three elk summer ranges by four predators along the eastern slopes of the Rocky Mountains, Alberta, Canada

	Summer range	Mean *P*_pred_	Mean *P*_elk_	Mean PR_scat_
Bear	West	0.43 ± 0.13	0.14 ± 0.11	0.21 ± 0.14
	YHT	0.24 ± 0.062	0.25 ± 0.11	0.20 ± 0.090
	East	0.19 ± 0.049	0.25 ± 0.21	0.16 ± 0.13
Cougar	West	0.58 ± 0.12	0.39 ± 0.18	0.24 ± 0.12
	YHT	0.57 ± 0.082	0.50 ± 0.21	0.29 ± 0.13
	East	0.63 ± 0.11	0.64 ± 0.31	0.43 ± 0.25
Coyote	West	0.73 ± 0.16	0.21 ± 0.112	0.17 ± 0.092
	YHT	0.80 ± 0.13	0.41 ± 0.218	0.35 ± 0.21
	East	0.78 ± 0.11	0.29 ± 0.174	0.24 ± 0.15
Wolf	West	0.21 ± 0.17	0.30 ± 0.17	0.062 ± 0.066
	YHT	0.34 ± 0.22	0.48 ± 0.29	0.22 ± 0.23
	East	0.11 ± 0.12	0.22 ± 0.26	0.037 ± 0.071

Note

Summer ranges include the Ya Ha Tinda (YHT), west of the YHT in Banff National Park, and east of the YHT on multiple use lands. All metrics were scaled between 0 and 1.

### 3.3 Landscape patterns: Kill site versus scat‐based predation risk

There was one clearly supported model (ΔAIC_c_ > 4), which included a positive effect of deciduous‐mixed forest land cover, herbaceous forage biomass, and open canopied areas, as well as a negative effect of distance to stream, terrain ruggedness and distance to forest edge (Table 5).

**TABLE 5:** Beta coefficients (β) and lower and upper confidence intervals (CI) for the top model (based on Akaike's Information Criterion corrected for small sample sizes, AIC_c_) for predicting locations of summer elk kill sites (PR_kill_) along the eastern slopes of the Rocky Mountains, Alberta, Canada, 2002–2016

Model variable	β	95% CI	
		Lower	Upper
Deciduous‐mixed forest	1.20	0.33	2.07
Distance to forest edge	2.09	0.87	3.32
Distance to streams	−7.24	−8.65	−5.82
Herbaceous forage biomass	3.78	2.58	4.98
Open cover	2.61	1.93	3.28
Terrain ruggedness	−6.67	−8.29	−5.05

**FIGURE 3:**
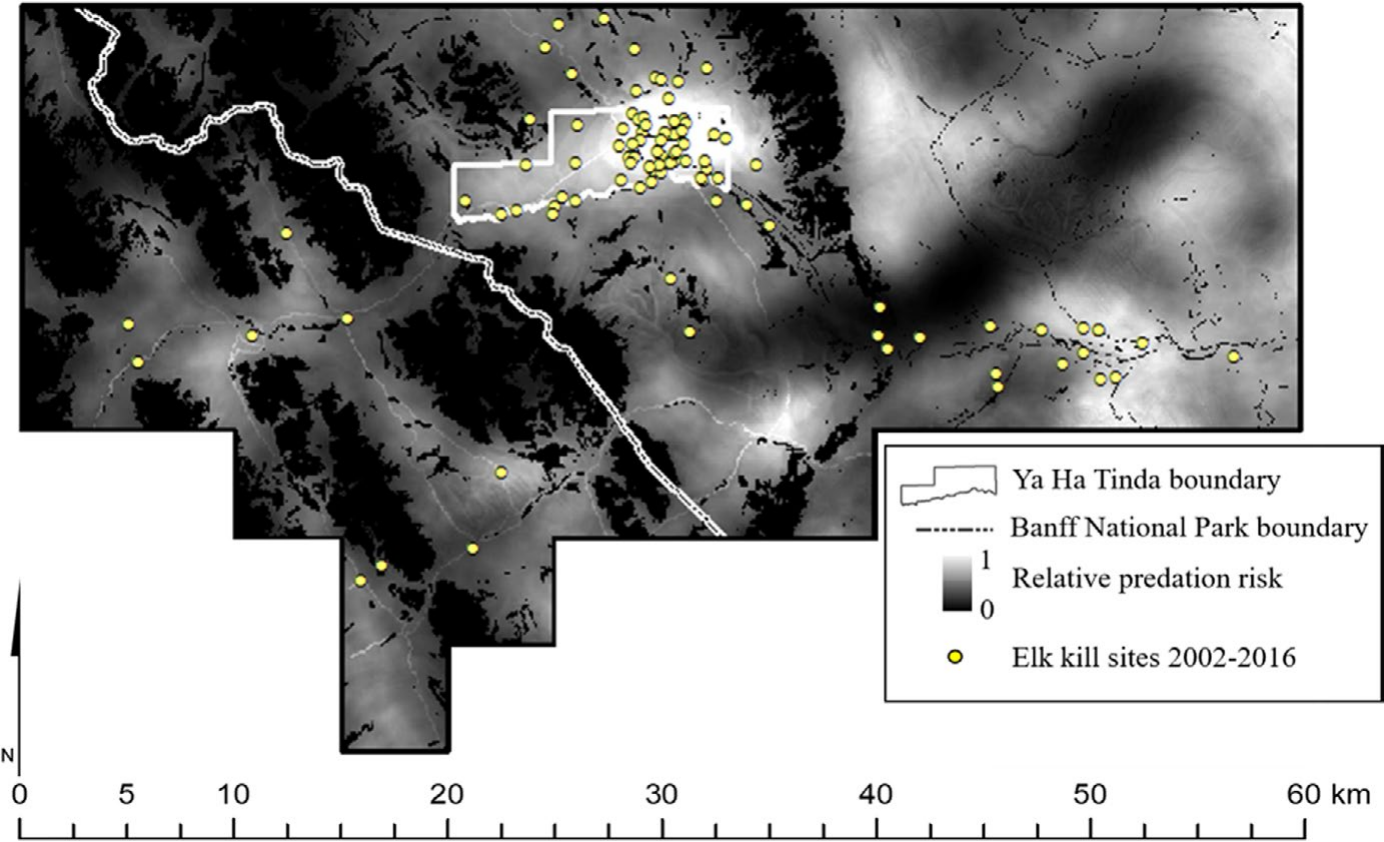
Predictions of total predation (PR_total_) risk for elk based on weighted predator resource selection (*P*_pred_) and elk presence in scats (*P*_elk_) summed across wolves, bear, cougars, and coyotes and scaled from 0 to 1 along the eastern slopes of the Rocky Mountains in Alberta, Canada. Yellow circles indicate locations of elk killed by bears, cougars, or wolves

**FIGURE 4:**
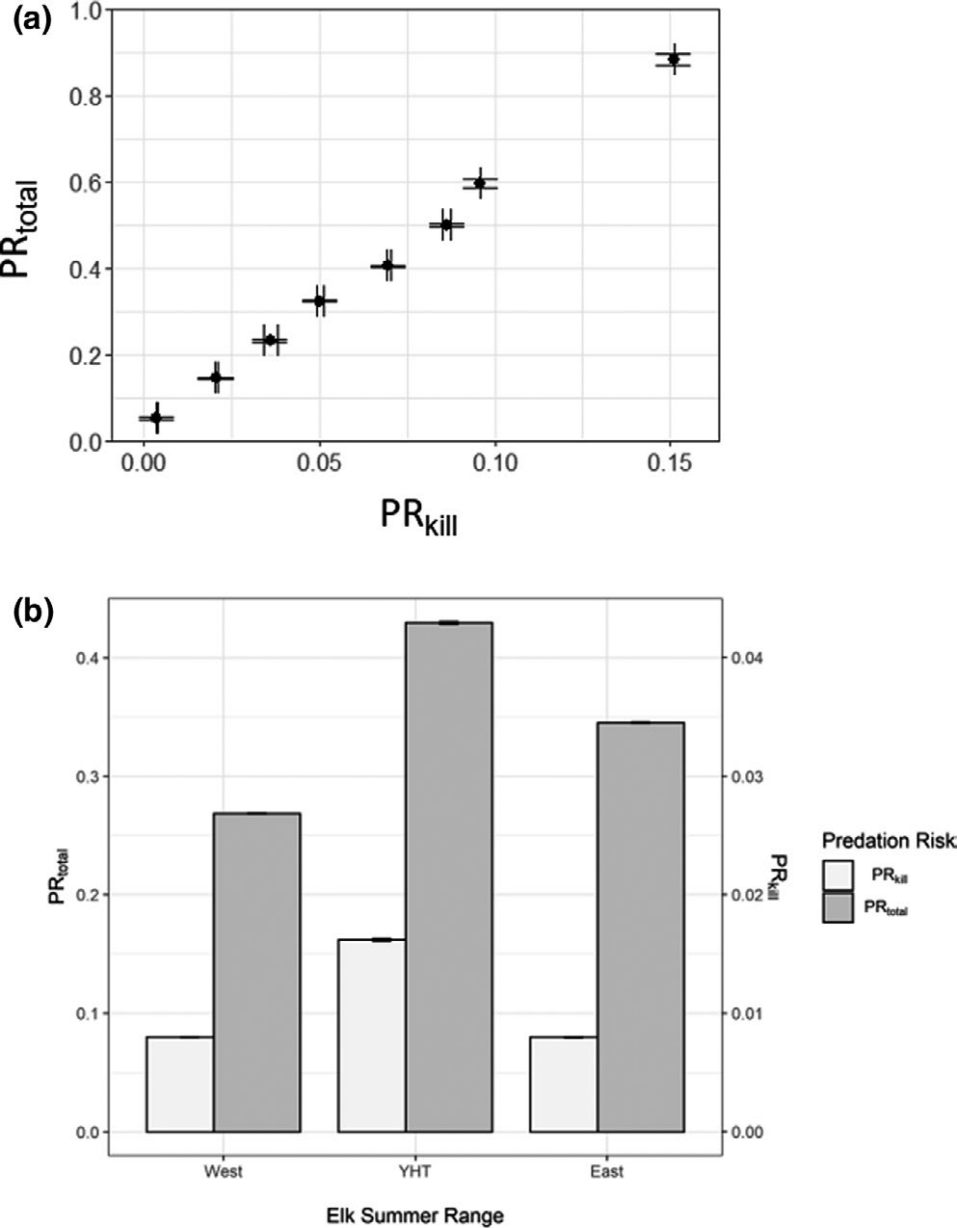
(a) Relationship between predicted kill‐based predation risk (PR_kill_) values and scat‐based total predation risk (PR_total_) values for elk from all 4 predators in the eastern slopes of the Rocky Mountains, Alberta, Canada. Values were binned (*n* = 10) based on equal bin width, and the mean value (± standard error) is presented. (b) Mean ± standard error PR_kill_ and PR_total_ for three elk summer ranges along the eastern slopes of the Rocky Mountains, Alberta, Canada

Predictions of total predation risk (PR_total_) based on scats generally corresponded to where we observed elk kills (Figure 3). When we quantified the relationship, we found the Spearman rank correlation between PR_total_ and PR_kill_ was high (*r_s_* = .98, *p* < .0001, *n* = 1000). We also found that mean PR_total_ and PR_kill_ summarized across the three spatial units representing elk summer ranges corresponded well (Figure 4a). Both indices indicated highest PR_total_ to elk from all predators occurred on Ya Ha Tinda (Figure 4b), which based on PR_scat_ resulted from moderate to high predation risk from all of the predators except cougars (Table 4).

## 4 DISCUSSION

Predictions of spatial risk based only on risk of predator encounter may not be sufficient to address some questions regarding prey anti‐predator behaviors (Robinson and Merrill, 2013), and this distinction is rarely acknowledged (Moll et al., 2017). Indeed, encounter‐only metrics could be misleading for mortality risk if different factors influence where prey are encountered versus where they are killed (Hebblewhite et al., 2005; Kauffman et al., 2007). We present a new method to quantify predation risk that incorporates where prey may encounter but also be killed. We found it produced predictions of predation risk comparable to those based on kill‐sites, the most common approach to quantify the risk of mortality (Kauffman et al., 2007; McKay et al., 2021; McPhee et al., 2012; Miller et al., 2015).

The scat‐based approach has distinct advantages over kill sites in that it is noninvasive, can be conducted over a relatively short period and early in a study, it can address multiple predator species at the same time, and it is relatively cost‐efficient when using trained dogs (Orkin et al., 2016; Wasser et al., 2004). For example, for wolf predation risk in Yellowstone National Park, prey kill‐site data were accumulated from winter aerial and ground surveys wolves over 10 years Kauffman et al., (2007), whereas in west‐central Alberta, Knopff et al. (2009) visited 1735 GPS telemetry clusters identified along movement paths of 24 GPS‐collared cougar, but only 37% of those clusters were locations where cougars killed prey. In this study, it took 14 years to accumulate 104 summer predator caused mortality events following >650 collared‐elk years.

At the same time, reliability of using fecal material to estimate predator distribution and prey mortality has been questioned for several reasons. First, false species identification of scats and prey contents of a scat can lead to inaccurate predictions (DeMatteo et al., 2018; Morin et al., 2016). For example, we found distinguishing between scats of bear species was more difficult based on visual inspection than anticipated and species misclassification was high (65%) when comparing based on DNA analysis (Spilker, 2019). Therefore, DNA analysis to verify species may be required to reduce uncertainty, which will increase the cost. Detecting the occurrence of prey in a scat using DNA analysis is also subject to error. For example, DNA analysis detected elk in 88% of scats where elk was detected macroscopically. The scats where elk was not detected through DNA analysis (i.e., 12%) were likely false negatives from PCR inconsistency (see Mumma et al., 2016). Numerous advances have been made in using DNA to identify scat contents, like high‐throughput sequencing (Di Bernardi et al., 2021; Pompanon et al., 2012) that may improve the accuracy but may not eliminate false negatives. It would be necessary to conduct captive feeding trials to adequately address factors affecting false negatives and decrease uncertainties in these analyses (see Thuo et al., 2019). The advanced methods were not as widely available when our study was designed in 2015, but future studies could benefit from such an approach. Because ~50% of the scats were macroscopically analyzed scats, the overall sample reduced the number of false negatives in the DNA‐analyzed scats (see above), but this method is also subject to uncertainty, especially when discriminating between hairs from closely related species like cervids (Kennedy & Carbyn, 1981).

Scat‐based predictions of predation risk also may not be appropriate where there is extensive scavenging by predators because scat contents reflect both kills and scavenging, whereas at kill sites criteria have been developed to distinguish when a carcass at a site is most likely a predator kill versus scavenging (e.g., Demski, 2015). In this study, the strong correlation between the scat‐based and kill‐site‐based metrics likely reflects minimal scavenging during summer for this suite of predators. In Yellowstone and Scandinavia, wolves were found to avoid bear kills and vice‐versa, particularly during the calving and early summer when bears target neonates and handling times are short relative to adult prey (Ordiz et al., 2020; Tallian et al., 2017). Although cougars may be displaced by wolves (Elbroch & Kusler, 2018; Kortello et al., 2007), cougar scavenging in an adjacent study area was found to be only one‐third as common in summer as in winter (Knopff et al., 2010). Coyotes avoid wolves and their kills particularly in summer (Klauder et al., 2021) and are known to be killed by wolves at carcasses (Merkle et al., 2009). Nevertheless, some caution should be exercised with using scat‐based approach in locations where scavenging is common because these trends are not universal (Bassi et al., 2018). Finally, dog training is key to maintaining efficient sampling to ensure a sufficient sample of scats are found. In this study, each of our dog‐handler teams were trained, and had a high probability (>.90) of detecting scats (Spilker, 2019). Where environmental factors hinder detection, it may be possible to adjust for missing scats by slowing the pace of dogs during the surveys.

A major consideration in developing and applying estimates of spatial risk is to appropriately match the approach to the spatial and temporal scales for the processes and questions addressed (Cusack et al., 2019; Moll et al., 2017; Prugh et al., 2019). For example, experimental approaches such as giving‐up densities (Altendorf et al., 2001) or interactions caught on remote cameras (Hernández et al., 2005) may be most appropriate to make site‐specific inferences of predation risk, whereas monitoring sequential movements of predators and prey simultaneously at very short temporal scales may lead to understanding how prey are successful in evasive tactics only under limited situations. Scat‐based approaches are likely best suited for assessing broad‐scale trends in predation as illustrated here. For example, because the exact date of scat deposition is unknown and surveys to collect sufficient number of scats occurred over a 12‐week summer season, we expected to gain little insight into predation risk dynamics within the summer season. Although scat locations reflect where a predator has been, they may not reflect where they spend the most time increasing the probability of prey encounter. However, we found that predator scats were associated with factors known to influence predator distribution in general. For example, bear scats were associated with areas of high forage quality (NDVI) and quantity (cut blocks), similar to models for grizzly bears where bears were associated with greenness and open canopy cover (Apps et al., 2004; Nielsen et al., 2002). Wolf scats were associated with flat areas (Hebblewhite & Merrill, 2007) and cougar scats were associated with areas of high forest edge (Atwood et al., 2009; Elbroch et al., 2013). We also found that predictions of high predation risk from the scat‐based RSFs corresponded well with predictions from telemetry‐based RSFs in the study area (Appendix S2).

Modeling where elk was found in scats compared to where scats were deposited (*P*_elk_) was intended to reflect additional features influencing where elk were more vulnerable or that availability of elk was high. However, the location of a scat containing elk being deposited at or some distance from an actual kill site depends on the movement and the rate of passage between consumption and defecation. For example, Webb et al., (2008) reported that 64% of the time wolves spent >8 h at a large‐prey site (including elk), and the geometric centers of clusters of GPS locations identified as kill sites were always within 200 m of actual kill locations. Because of this uncertainty, we used species‐specific, spatial domains (1.5–3 km) to assess *P*_elk_ that were a compromise between movement and passage rates (Appendix S1). Indeed, outcomes of the models predicting *P*_elk_ are consistent with where we would expect elk to show high use or to be vulnerable. For example, elk select for areas with abundant forage biomass in summer (Berg et al., 2021; Hebblewhite & Merrill, 2009), which is where we found a higher probability of elk being present in the scat of all species. Elk were less commonly found in bear scats in open areas where they may detect predators more easily, and more often in cougar scats along forest edges where prey are reported be vulnerable to cougars (Holmes & Laundré, 2006; Laundré & Hernández, 2003); further, elk were found in wolf scats associated with rugged terrain, which is where Torretta et al. (2018) found elk kill sites.

Evidence that a scat‐based approach can describe broad‐scale patterns of predation risk is supported by the correspondence between scat‐based predation risk on the summer ranges of the Ya Ha Tinda elk population and previously described patterns of telemetry‐based estimates of predation risk (Berg, 2019; Hebblewhite et al., 2018). Using specific locations of collared female elk, Hebblewhite and Merrill (2009) reported that elk migrating into Banff National Park to summer in the early 2000s were exposed to lower wolf predation risk (derived from telemetry‐based RSFs weighted by pack size) than elk at the Ya Ha Tinda, and this pattern was reaffirmed at the scale of an elk's home range in 2013–2016 (Berg et al., 2021). Although no previous studies in the area addressed predation risk to elk from cougars, home ranges of eastern migrant elk had higher forest edge due to forestry activity (Berg et al., 2021), where cougars successfully hunt and stalk their prey (Holmes & Laundré, 2006; Laundré & Hernández, 2003). Berg et al. (2021) also reported that bear predation risk (derived telemetry‐based RSFs weighted by abundance) was higher in the summer ranges of western migrants than either resident elk at the Ya Ha Tinda or eastern migrants, which is consistent with the results presented here. However, the pattern in elk occurring in bear scats did not follow the same spatial pattern; instead, mean *P*_elk_ of western migrant ranges was lower than in residents ranges at the Ya Ha Tinda, such that overall predation risk (*P*_scat_) was similar between these two areas (Table 5).

The above is an example of where an encounter‐only‐based model of bear predation risk might be a misleading index for mortality risk and points to the need to strengthening the link between risk of predation, and kill or predation rates as a key next step for addressing questions in spatial predator‐prey dynamics. For example, Hebblewhite et al. (2018) reported that in this area bear predation, unlike wolf predation, is density‐dependent and western migrant elk have declined faster than resident elk since 2002 (Hebblewhite et al., 2006). Finally, our evaluation of the scat‐based spatial patterns of predation is based primarily on what we know about predation on female elk, yet elk in scats may also reflect mortality of male elk. While it may be possible to amplify nuclear DNA to elicit the sex of prey consumed from scats in fish species (Balbag et al., 2019), we are not aware of any methods to successfully do this for terrestrial predators. This merits further study because Martin (2021) found that selection by males was associated less with predation risk than forage compared to females, and that cause‐specific mortality differed between collared female and male elk.

## 5 CONCLUSION

We illustrate a new approach for estimating broad‐scale predation risk to prey based on distribution and contents of predator scats and found it corresponds well with the results of using kill sites of adult females and calves that also include components of encounter and mortality. It has the advantage of being able to distinguish components of predation, such as where prey may encounter predators and where they are killed, which may provide insights into the dynamics of predation. It can be used to sample broad areas cost‐effectively over a relatively short time frame when using detection dogs to get a snapshot of spatial predation risk, which lends itself to repeat sampling for detecting changes in spatial risk in the same area over time. Considering the scavenging context, our approach may not be feasible if the study objective is to assess predator kill rates. As with other methods, appropriate sampling design and reducing uncertainty with observer training (e.g., dogs and handlers) and auxiliary data such as DNA to confirm species identification will be key considerations.

## CONFLICT OF INTEREST

The authors declare that they have no known competing financial interests or personal relationships that could have appeared to influence the work reported in this paper.

## AUTHOR CONTRIBUTION

**Kara M. MacAulay:** Conceptualization (equal); Data curation (equal); Formal analysis (lead); Funding acquisition (equal); Investigation (equal); Methodology (lead); Project administration (equal); Resources (lead); Validation (lead); Visualization (lead); Writing – original draft (lead); Writing – review & editing (lead). **Eric G. Spilker:** Conceptualization (equal); Data curation (equal); Formal analysis (supporting); Funding acquisition (equal); Investigation (equal); Methodology (supporting); Project administration (equal); Resources (supporting); Visualization (supporting); Writing – original draft (supporting); Writing – review & editing (supporting). **Jodi E. Berg:** Conceptualization (supporting); Data curation (supporting); Funding acquisition (equal); Investigation (supporting); Methodology (supporting); Writing – review & editing (supporting). **Mark Hebblewhite:** Conceptualization (supporting); Funding acquisition (equal); Investigation (supporting); Supervision (supporting); Writing – review & editing (supporting). **Evelyn H. Merrill:** Conceptualization (equal); Formal analysis (supporting); Funding acquisition (equal); Methodology (supporting); Project administration (equal); Resources (supporting); Supervision (lead); Validation (supporting); Visualization (supporting); Writing – original draft (supporting); Writing – review & editing (supporting).

## Supporting information

Appendix S1

Appendix S2

Appendix S3

Appendix S4

Appendix S5

Appendix S6

## Data Availability

Global Positioning System (GPS) data are available on the MOVEBANK data repository (associated with Movebank project ID 72264071; data repository https://doi.org/10.5441/001/1.k8s2g5v7, Hebblewhite & Merrill, 2016). Processed datasets used for the scat and kill site spatial analyses are available from the Dryad Digital Repository: https://doi.org/10.5061/dryad.2ngf1vhpv, MacAulay et al., 2022).
