# Supplementary material for: Beyond the encounter: Predicting multi‐predator risk to elk (Cervus canadensis) in summer using predator scats
Source: Ecol Evol. 2022 Feb 14;12(2):e8589. doi: 10.1002/ece3.8589 (PMC8843817; doi:10.1002/ece3.8589)
Supplement: Supplementary file 1 — Appendix S1 [file ECE3-12-e8589-s001.docx]

| Appendix S1. Summary of movement rates (km per day) and digestive retention times (hr) used to estimate the size (km) of buffers used to quantify the environmental variables around each scat of each predator species in the eastern slopes of the Rocky Mountains, Alberta, Canada, 2013 – 2016. Buffers were used in canonical correspondence analysis and spatial risk analysis. | | | | | | | |
| --- | --- | --- | --- | --- | --- | --- | --- |
|  | Movement per  day (km) | Citation | Digestive retention time (hours) | Citation | Minimum buffer size | Maximum buffer size | Buffer Radius for Analysis (km) |
| Grizzly bear | 2.4 – 3.4 | Craighead 1976; Gibeau et al. 2001 | 13.0 ± 2.3 | Pritchard and Robbins 1990; Elfström et al. 2013 | 2.4 km/day*0. days = 1.3 | 3.4 km/day * 0.54 days = 1.8 km | 1.5 |
| Black bear | 1.0 – 1.7 | Amstrup and Beecham 1976; Garshelis 1978; Garshelis et al. 1983 | 12.9 ± 3.0 | Pritchard and Robbins 1990 | 1.0 km/day * 0.54 days = 0.54 | 1.7 km/day * 0.54 days = 0.90 km |  |
| Wolf | 4.4 – 6.0 | Jedrzejewski et al. 2002; Webb 2009 | 8 – 56 | Floyd et al. 1978 | 4.4 km/day * 0.3 days = 1.3 | 4.4 km/day * 2.3 days = 10 km | 3 |
|  |  |  |  |  | 6 km/day * 0.3 days = 1.8 km | 6 km/day * 2.3 days = 13.8 km |  |
|  |  |  |  |  | average = 1.5 km | average = 12 km |  |
| Coyote | 4 – 4.4 (straight line distance) | Bekoff 1977; Andelt and Gipson 1979 | 2 – 5 km (bead “retention” distance)^a^ | Lunney et al. 2002 | 2 km | 5 km | 2 |
| Cougar | 10.4 - 16.5 | Laundré 2005; Dickson and Beier 2007 | 0.4-4.2 km from kill site^b^ | Beier et al. 1995 | 0.4 km | 4.2 km | 2 |
| ^a^ Gut passage time for coyotes could not be found in the literature. We used a measure of “retention distance” calculated for foxes (*Vulpes Vulpes*) and dogs (*Canis lupus familiaris*) using non-poisoned baits containing bead markers and measuring the distance from marked scat to the bait site.  ^b^ Gut passage time for cougars could not be found in literature. We used a measure of “retention distance” calculated for cougars where the authors reported an average distance of cougar scat from the last kill site. | | | | | | | |
