# Supplementary material for: Beyond the encounter: Predicting multi‐predator risk to elk (Cervus canadensis) in summer using predator scats
Source: Ecol Evol. 2022 Feb 14;12(2):e8589. doi: 10.1002/ece3.8589 (PMC8843817; doi:10.1002/ece3.8589)
Supplement: Supplementary file 2 — Appendix S2 [file ECE3-12-e8589-s005.docx]

| **Appendix S2.** Summary of model selection results based on AIC for resource utilization functions (RUF) that predict elk utilization based on habitat and predation variables (wolf = wolf RSF, gb = grizzly bear RSF). The top RUF (bolded) was used to predict the resource utilization values in the study area along the eastern slopes of the Rocky Mountains in Alberta, Canada. Variables defined in Table 1. | | | | |
| --- | --- | --- | --- | --- |
| Model Variables | k | AIC | ΔAIC | weight |
| **Herbfg + burn + wolf - gb - distedge** | **6** | **9565.66** | **0.00** | **1.00** |
| Totalfg + burn + wolf - gb - distedge | 6 | 9780.10 | 214.44 | 0.00 |
| Wolf – gb | 3 | 9789.52 | 223.86 | 0.00 |
| Herbfg + burn - gb - distedge | 5 | 9830.78 | 265.12 | 0.00 |
| Herbfg + burn – distedge | 4 | 9977.18 | 411.52 | 0.00 |
| Null model | 2 | 10020.31 | 454.65 | 0.00 |
| Herbfg + burn + wolf - distedge | 5 | 10075.38 | 509.72 | 0.00 |
