# Supplementary material for: Beyond the encounter: Predicting multi‐predator risk to elk (Cervus canadensis) in summer using predator scats
Source: Ecol Evol. 2022 Feb 14;12(2):e8589. doi: 10.1002/ece3.8589 (PMC8843817; doi:10.1002/ece3.8589)
Supplement: Supplementary file 3 — Appendix S3 [file ECE3-12-e8589-s003.docx]

| Appendix S3. Summary of model selection for scat-based predator resource selection functions in the eastern slopes of the Rocky Mountains, Alberta, Canada, 2014 – 2016. Top RSFs shown in bold. Variables defined in Table 1. | | | |
| --- | --- | --- | --- |
| Model variables^a^ | k | AIC_c_ | ΔAIC_c_ |
| Bear |  |  |  |
| -Conifer+ndvi+slope+trailuse+distroad+trailuse*distroad | 6 | 1462 | 0 |
| -C**onifer+ndvi+slope+trailuse+distroad+cutdens+trailuse*distroad** | 7 | 1465 | 3 |
| -Conifer+cutdens+ndvi+slope+trailuse+distroad | 5 | 1466 | 4 |
| Full model^a^ | 13 | 1483 | 21 |
| Null model | 0 | 1608 | 146 |
| Wolf |  |  |  |
| **-Distwater-slope-cutdens+trailuse+distroad** | 5 | **1637** | **0** |
| -Distwater-slope-cutdens+trailuse+distroad+edgedens | 6 | 1639 | 2 |
| -Distwater-slope-cutdens+trailuse+distroad-herb | 6 | 1639 | 2 |
| -Distwater-slope-cutdens+trailuse+distroad+edge-herb | **7** | 1642 | 4 |
| -Distwater-slope+trailuse+distroad | 4 | 1647 | 10 |
| Full model^a^ | 13 | 1653 | 16 |
| Null model | 0 | 1844 | 207 |
| Coyote |  |  |  |
| **Shrub-slope+trailuse+distroad** | **4** | **1041** | **0** |
| Shrub, edgedens, -slope+trailuse+distroad | 5 | 1041 | 0 |
| Shrub, -slope+trailuse+distroad-herb | 5 | 1041 | 0 |
| Shrub, edge, -slope+trailuse+distroad-herb | 6 | 1042 | 1 |
| Edgedens, -slope+trailuse+distroad-herb | 5 | 1043 | 2 |
| -Slope+trailuse+distroad | 3 | 1044 | 3 |
| -Slope+trailuse+distroad-trailuse*distroad | 4 | 1046 | 5 |
| Full model^a^ | 13 | 1053 | 12 |
| Null model | 0 | 1163 | 122 |
| Cougar |  |  |  |
| **-Conifer+edgedens** | **2** | **355** | **0** |
| -Conifer | 1 | 358 | 3 |
| -Conifer+edgedens+shrub-rugg | 4 | 359 | 4 |
| Null model | 0 | 360 | 5 |
| Edgedens | 1 | 361 | 6 |
| Edgedens+rugg | 2 | 362 | 7 |
| Full model^a^ | 13 | 375 | 20 |
| ^a^Full model: ± elev ± slope ± conifer ± mixed ± shrub ± herb ± edge ± fire ± cutdens ± ndvi ± distwater ± distroad ± trailuse | | | |
