# Supplementary material for: Beyond the encounter: Predicting multi‐predator risk to elk (Cervus canadensis) in summer using predator scats
Source: Ecol Evol. 2022 Feb 14;12(2):e8589. doi: 10.1002/ece3.8589 (PMC8843817; doi:10.1002/ece3.8589)
Supplement: Supplementary file 4 — Appendix S4 [file ECE3-12-e8589-s002.docx]

**Appendix S4:** Evaluating scat-based RSF predictions (*P*_pred_)

We evaluated the spatial prediction of scat-based wolf and bear RSF resource selection functions by comparing them to RSF values derived from locations of GPS-collared wolves (Hebblewhite & Merrill, 2007) and from grizzly bears ~200 km north of our study area (Nielsen et al., 2002). We used Spearman rank correlations at a random set of points (*n* = 1,000) distributed across the study area after removing areas of high elevations (> 2,000m), bare rock, or ice. We took the average of telemetry-based wolf RSF values from a previously developed RSF model for day and night resource selection by 15 GPS-collared wolves in five packs for summers 2002 – 2004 (Hebblewhite & Merrill 2007). The telemetry-based grizzly bear RSFs were derived from bears (*n* = 9: 6 females, 3 males; Nielsen et al., 2002) for the seasons of hypophagia (15 April – 14 June), early hyperphagia (15 June – 7 August), and late hyperphagia (8 August to denning) in 2004. Telemetry-based grizzly bear and wolf RSF values were scaled from 0 – 1, then aggregated into 10 classes based on approximately equal area representation (Nielsen et al., 2002). We updated both wolf and bear telemetry-based RSFs for years 2013 – 2015 to account for landcover changes (such as new timber harvest and fires, MacAulay 2019). We then predicted values for a 30 x 30-m cell were a weighted average based on days in each bear season (*i.e.*, 46 days in hypophagia, 45 in early hyperphagia, and 77 days in late hyperphagia). There was no available telemetry-based black bear RSF, but we estimated about 85% of bear scats used in the scat-based RSF were from grizzly bears due to the DNA results from a subsample collected (Spilker, 2019).

Scat-based and telemetry-based RSF values were correlated for wolves (*r_s_* = 0.18, *P* < 0.001, *n* = 1,000) and bears, depending on season (hypophagia, early hyperphagia, and late hyperphagia. See Nielsen et al. 2002; *r_s_* = 0.17-0.25, *P* < 0.001), but the relationships were not strongly linear (Figure S2). Nevertheless, scat-based values increased as telemetry-based values increased. When aggregated into 10 bins, rank correlations of the mean RSF bin values indicated much higher correspondence (wolf: *r_s_* = 0.92, *P* < 0.0001; grizzly bear: *r_s_* = 0.68, *P* < 0.0001).


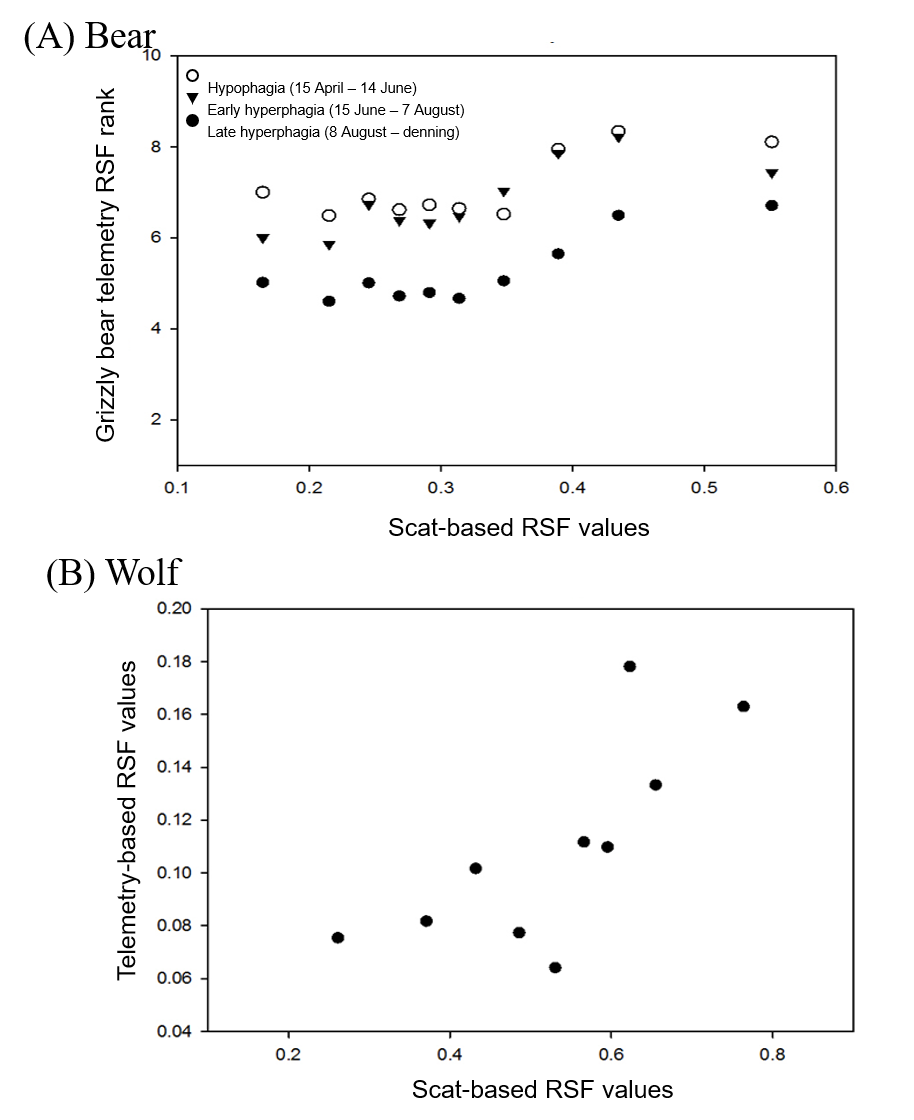


Figure S2. Relationships between mean predicted scat-based resource selection function (*P*_pred_) values and mean predicted telemetry-based RSF values for (a) bears and (b) wolves in the eastern slopes of the Rocky Mountains, Alberta, Canada, 2013 – 2016. Binning has smoothed the data to allow for sufficient characterization of the nature of the relationship between increasing RSF values between models.
