# Supplementary material for: Beyond the encounter: Predicting multi‐predator risk to elk (Cervus canadensis) in summer using predator scats
Source: Ecol Evol. 2022 Feb 14;12(2):e8589. doi: 10.1002/ece3.8589 (PMC8843817; doi:10.1002/ece3.8589)
Supplement: Supplementary file 5 — Appendix S5 [file ECE3-12-e8589-s004.docx]

| Appendix S5. Summary of model selection results for predicting elk presence in scats for four predators (*P*_elk_) along the eastern slopes of the Rocky Mountains, Alberta, Canada, 2013 – 2016. Top models used to predict *P*_elk_ shown in bold. Variables defined in Table 1. | | | | | |
| --- | --- | --- | --- | --- | --- |
| Model variables^a^ | k | AIC | ΔAIC | Weight | |
| Bear |  |  |  |  | |
| Herbfg - open - disttrail | 4 | 151.54 | 0.00 | 0.47 | |
| **Herbfg - open** | **3** | **152.99** | **1.45** | **0.23** | |
| Herbfg - open - rugg | 4 | 153.97 | 2.44 | 0.14 | |
| herbfg - open - roaddens | 4 | 155.02 | 3.49 | 0.08 | |
| RUF - open + rugg | 4 | 157.03 | 5.50 | 0.03 | |
| -Open - disttrail | 3 | 157.20 | 5.66 | 0.03 | |
| Herbfg - disttrail | 3 | 157.44 | 5.90 | 0.02 | |
| Herbfg | 2 | 160.31 | 8.77 | 0.01 | |
| Null | 1 | 166.00 | 14.46 | 0.00 | |
| -Open | 2 | 167.07 | 15.53 | 0.00 | |
| Coyote | | | | | |
| **Herbfg + distwater - roaddens** | **4** | **217.03** | **0.00** | **0.71** | |
| Herbfg + distwater | 3 | 220.51 | 3.49 | 0.12 | |
| Herbfg - roaddens | 3 | 220.57 | 3.54 | 0.12 | |
| Herbfg | 2 | 222.30 | 5.27 | 0.05 | |
| Distwater | 2 | 231.61 | 14.58 | 0.00 | |
| Distwater - roaddens | 3 | 231.80 | 14.77 | 0.00 | |
| Null | 1 | 234.39 | 17.37 | 0.00 | |
| RUF | 2 | 235.06 | 18.04 | 0.00 | |
| -Roaddens | 2 | 235.76 | 18.74 | 0.00 | |
| Cougar | | | | | |
| Disttrail | 2 | 33.39 | 0.00 | 0.30 | |
| **Edgedens** | **2** | **33.67** | **0.28** | **0.26** | |
| Edgedens + disttrail | 3 | 34.18 | 0.79 | 0.20 | |
| Edgedens + herbfg | 3 | 35.04 | 1.64 | 0.13 | |
| Edgedens + disttrail - RUF | 4 | 36.34 | 2.95 | 0.07 | |
| Null | 1 | 38.66 | 5.27 | 0.02 | |
| -RUF | 2 | 40.75 | 7.36 | 0.01 | |
| Wolf |  |  |  |  |  |
| **Herbfg + rugg - decid** | **4** | **288.93** | **0.00** | **0.43** |  |
| Herbfg + rugg - burn - decid | 5 | 289.71 | 0.79 | 0.29 |  |
| Herbfg + rugg | 3 | 305.06 | 16.13 | 0.00 |  |
| Herbfg - decid | 3 | 317.68 | 28.75 | 0.00 |  |
| -Decid - rugg + RUF | 4 | 370.69 | 81.76 | 0.00 |  |
| -Burn - decid - rugg + RUF | 5 | 372.26 | 83.33 | 0.00 |  |
| -Decid - rugg | 3 | 385.86 | 96.93 | 0.00 |  |
| -Burn - decid - rugg | 4 | 387.89 | 98.96 | 0.00 |  |
