# Supplementary material for: Beyond the encounter: Predicting multi‐predator risk to elk (Cervus canadensis) in summer using predator scats
Source: Ecol Evol. 2022 Feb 14;12(2):e8589. doi: 10.1002/ece3.8589 (PMC8843817; doi:10.1002/ece3.8589)
Supplement: Supplementary file 6 — Appendix S6 [file ECE3-12-e8589-s006.docx]

| Table S6. Summary of model selection results for predicting sites of elk kills by bears, cougars, and wolves (PR_kill_) along the eastern slopes of the Rocky Mountains, Alberta, Canada, 2002 – 2016. The top model based on Akaike’s Information Criterion corrected for small sample sizes (AIC_c_) is shown in bold. | | | | | | | |
| --- | --- | --- | --- | --- | --- | --- | --- |
| Model variables^a^ | k | | AICc | ΔAICc | Weight | | |
| **Decid + distedge - distwater + herbfg + open - rugg** | | **7** | **2075** | **0.00** | | **0.82** |  |
| Distedge - distwater + herbfg + open - rugg | | 6 | 2079 | 4.64 | | 0.08 |  |
| Decid - distwater + herbfg + open - rugg | | 6 | 2083 | 8.25 | | 0.08 |  |
| - Distwater + herbfg + open - rugg | | 5 | 2086 | 11.97 | | 0.01 |  |
| Decid + distedge - distwater + open - rugg | | 6 | 2112 | 37.50 | | 0.00 |  |
| Decid - distwater + open - rugg | | 5 | 2118 | 42.83 | | 0.00 |  |
| Decid + distedge - distwater + herbfg - rugg | | 6 | 2133 | 57.99 | | 0.00 |  |
| Distedge - distwater + open - rugg | | 5 | 2144 | 69.21 | | 0.00 |  |
| - Distwater + open - rugg | | 4 | 2148 | 73.51 | | 0.00 |  |
| Decid + distedge - distwater + herbfg + open | | 6 | 2160 | 85.34 | | 0.00 |  |
| Decid + distedge + herbfg + open - rugg | | 6 | 2229 | 154.17 | | 0.00 |  |
| ^a^Variables defined in Table 1. |  | |  |  | |  |  |
